# Modulation of cerebral endothelial cell function by TGF-β in glioblastoma: VEGF-dependent angiogenesis versus endothelial mesenchymal transition

**DOI:** 10.18632/oncotarget.4310

**Published:** 2015-06-15

**Authors:** Shanmugarajan Krishnan, Emese Szabo, Isabel Burghardt, Karl Frei, Ghazaleh Tabatabai, Michael Weller

**Affiliations:** ^1^ Laboratory of Molecular Neuro-Oncology, Department of Neurology and Neuroscience Center, University Hospital and University of Zurich, Zurich, Switzerland; ^2^ Laboratory of Molecular Neuro-Oncology, Department of Neurosurgery and Neuroscience Center, University Hospital and University of Zurich, Zurich, Switzerland; ^3^ Interdisciplinary Division of Neuro-Oncology, Departments of Vascular Neurology and Neurosurgery, University Hospital Tübingen, Tübingen, Germany

**Keywords:** angiogenesis, glioblastoma, TGF-β, VEGF, PlGF

## Abstract

Glioblastoma are among the most angiogenic tumors. The molecular mechanisms that control blood vessel formation by endothelial cells (EC) in glioblastoma remain incompletely understood. Transforming growth factor-β (TGF-β) is a key regulatory cytokine that has proinvasive and stemness-maintaining autocrine properties in glioblastoma and confers immunosuppression to the tumor microenvironment. Here we characterize potential pro- and anti-angiogenic activities of TGF-β in the context of glioblastoma *in vitro*, using human brain-derived microvascular endothelial cells (hCMEC/D3) and glioblastoma-derived endothelial cells (GMEC) as model systems. We find that TGF-β induces vascular endothelial growth factor (VEGF) and placental growth factor (PlGF) mRNA expression and protein release in a TGF-β receptor (TβR) II / activin-like kinase (ALK)-5-dependent manner under normoxia and hypoxia, defining potential indirect proangiogenic activity of TGF-β in glioblastoma. In parallel, exogenous TGF-β has also inhibitory effects on EC properties and induces endothelial-mesenchymal transition (EndMT) in hCMEC and GMEC. Accordingly, direct inhibition of endogenous TGF-β/ALK-5 signalling increases EC properties such as tube formation, von-Willebrand factor (vWF) and claudin (CLDN) 5 expression. Yet, the supernatant of TGF-β-stimulated hCMEC and GMEC strongly promotes EC-related gene expression and tube formation in a cediranib-sensitive manner. These observations shed light on the complex pro- and anti-angiogenic pathways involving the cross-talk between TGF-β and VEGF/PLGF signalling in glioblastoma which may involve parallel stimulation of angiogenesis and EndMT in distinct target cell populations.

## INTRODUCTION

Blood supply is an essential prerequisite for solid tumor growth and therefore a *bona fide* target for intervention. The processes by which tumors initiate and maintain their blood supply are complex and have remained incompletely understood. Vasculogenesis is defined as the initial migration and differentiation of endothelial precursor cells to form blood vessels *de novo*. Subsequent to this, angiogenesis occurs by the formation of new blood vessels from pre-existing ones, also termed sprouting angiogenesis. Angiogenesis in glioblastoma is characterized by a neovasculature that has an erratic basement membrane, reduced pericyte coverage, decreased astrocytic end feet association with blood vessels, and altered expression of adhesion molecules on endothelial cells (EC) [1]. This abnormal vascularization causes excessive leakiness and promotes edema formation.

One traditional view states that hypoxic tumor cells release vascular endothelial growth factor (VEGF) which in turn counteracts tumor hypoxia by mediating the growth of new blood vessels. However, it is unlikely that all blood vessel formation in glioblastoma is triggered by hypoxia-mediated VEGF signaling, notably early in the disease course when there is probably little hypoxia. Thus, alternative pathways regulating tumor-associated angiogenesis beyond hypoxia-driven VEGF synthesis are likely to exist.

Transforming growth factor (TGF)-β is a prototype member of a cytokine family involved in most cancer-associated processes. In glioblastoma, immunosuppression and autocrine control of migration, invasiveness and stemness have received most attention. However, TGF-β has also been implicated in angiogenesis in various model systems including physiological and pathological angiogenesis. Direct TGF-β signalling on endothelial cells has been linked mainly to two type I receptors, activin-like receptor kinases (ALK)-1 and ALK-5. These two TGFβR I-mediated pathways have been attributed to opposing effects in response to TGF-β: ALK-1 on interaction with TβRII induced Smad 1/5 phosphorylation to induce endothelial cell proliferation [2] and migration [3] whereas ALK-5 recruited by TβRII induces Smad 2/3 phosphorylation, thereby inhibiting the above-mentioned effects [3]. TGF-β family ligands can interact with co-receptors represented by endoglin (CD105), which is highly expressed in proliferating endothelial cells, and betaglycan (TβRIII). In the present study, we investigated the interaction and dependence of these key signaling pathways and their role for glioma angiogenesis, using hCMEC and GMEC as models.

## RESULTS

### hCMEC express TGF-β receptors and coreceptors

**Figure 1:**
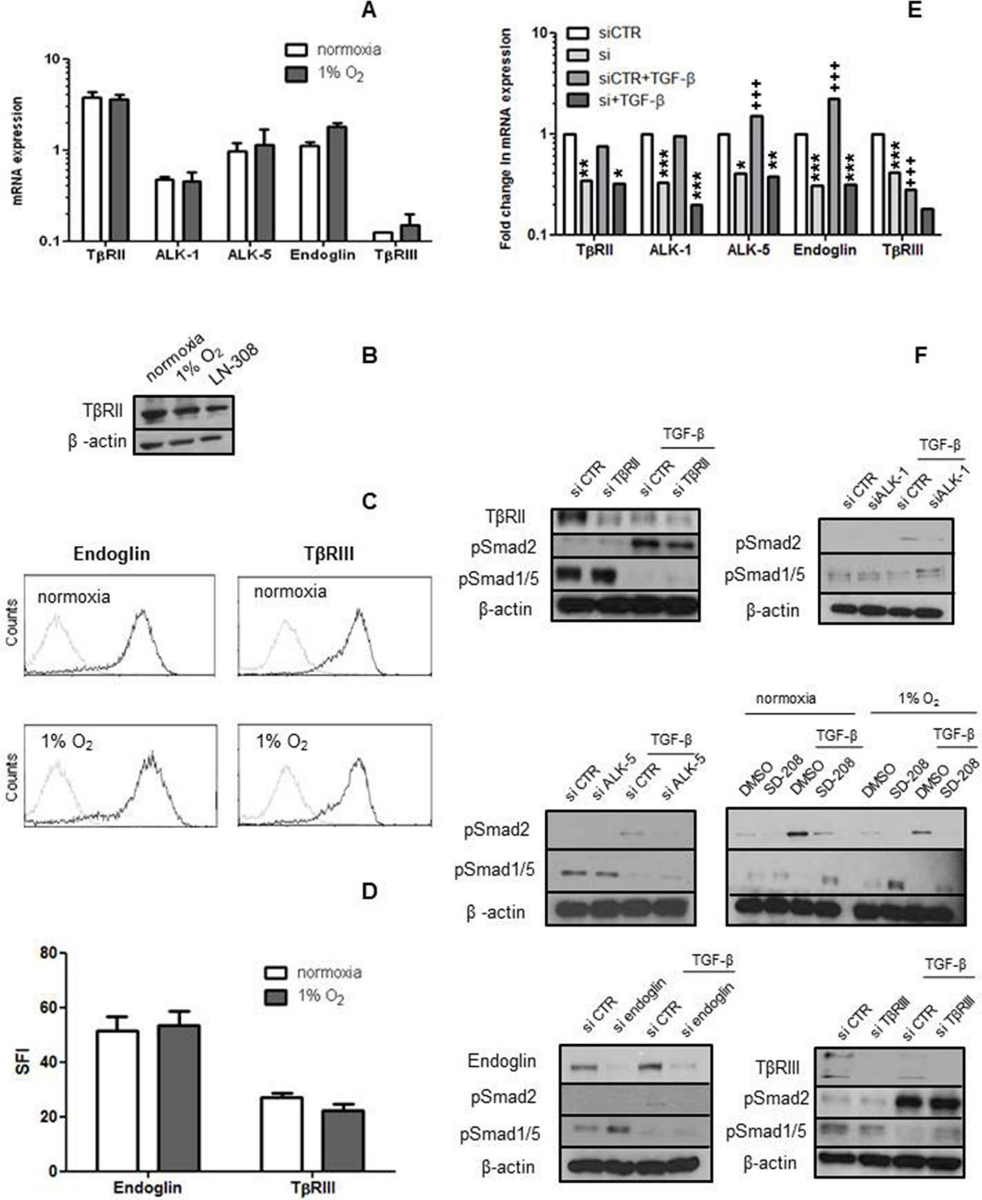
hCMEC express TGF-β receptors and coreceptors **A–D.** The levels of mRNA for TGF-β receptors RII, ALK-1, ALK-5 and endoglin or RIII were assessed by RT-PCR (A) and protein by immunoblot (B) or flow cytometry (C, D) as feasible under normoxia and hypoxia. Data in A are expressed as mean values and SD (*n* = 4). **E, F.** TGF-β receptor gene silencing was verified on mRNA (*n* = 3) and protein level. In F, the effect of TGF-β receptor gene silencing on constitutive and TGF-β (10 ng/ml, 72 h)-evoked pSmad2 and pSmad1/5 levels was studied by immunoblot. SD-208 (1 μM, 72 h) was included to confirm the ALK-5 findings (*n* = 3). (*p < 0.05, **p < 0.01, ***p < 0.001, one-way ANOVA followed by Tukey's post hoc test, effect of specific siRNA, ^+++^*p* < 0.001, effect of TGF-β).

We first analysed whether TGF-β receptors *(*TβRII, ALK-1 and ALK-5) and co-receptors (endoglin and TβRIII) were expressed at mRNA and protein level in hCMEC. All receptors were expressed at mRNA level under normoxia and hypoxia, without significant differences (*p* > 0.05) (Figure 1A). TβRII, endoglin and TβRIII protein levels were also unaltered in hypoxia (Figure 1B–1D). Hypoxia decreased pSmad1/5 and Smad5 levels, but did not affect pSmad2, Smad2, Smad1 or secreted levels of TGF-β. Like most non-neoplastic cells, hCMEC released more unprocessed than active TGF-β (Supplementary Figure 1A).

After confirming the expression of the TGF-β receptors and ligand, we transiently silenced TβRII, ALK-1, ALK-5, endoglin or TβRIII in the absence or presence of TGF-β. Silencing was confirmed at the mRNA and protein levels wherever feasible (Figure 1E–1F). The specificity of silencing ALK-1 versus ALK-5 was verified by RT-PCR (Supplementary Figure 1B–1C). Interestingly, exposure to exogenous TGF-β decreased TβRIII and increased ALK-5 at mRNA level and endoglin expression at mRNA and protein levels. Constitutive pSmad2 was faintly detectable and not induced by receptor silencing. Constitutive pSmad1/5 levels increased mildly with silencing of endoglin, but not with silencing of the other receptors (Figure 1E–1F). TGF-β strongly induced pSmad2, and this induction was attenuated by the silencing of TβRII, ALK-1, ALK-5 or endoglin, although not TβRIII. TGF-β strongly decreased pSmad1/5 levels, and this effect was attenuated somewhat by the silencing of ALK-1, ALK-5, TβRII, TβRIII or endoglin, but robustly by SD-208. TβRII levels were strongly reduced at 72 h after exposure to TGF-β (Figure 1F).

Under these experimental conditions, exposure of hCMEC to TGF-β did not induce significant loss of viability assessed by trypan blue dye exclusion, cell cycle arrest assessed by PI staining or features of apoptosis as assessed by annexin V / propidium labeling (Supplementary Figure 2).

### TGF-β induces VEGF mRNA expression and protein release in hCMEC under normoxic and hypoxic conditions

**Figure 2:**
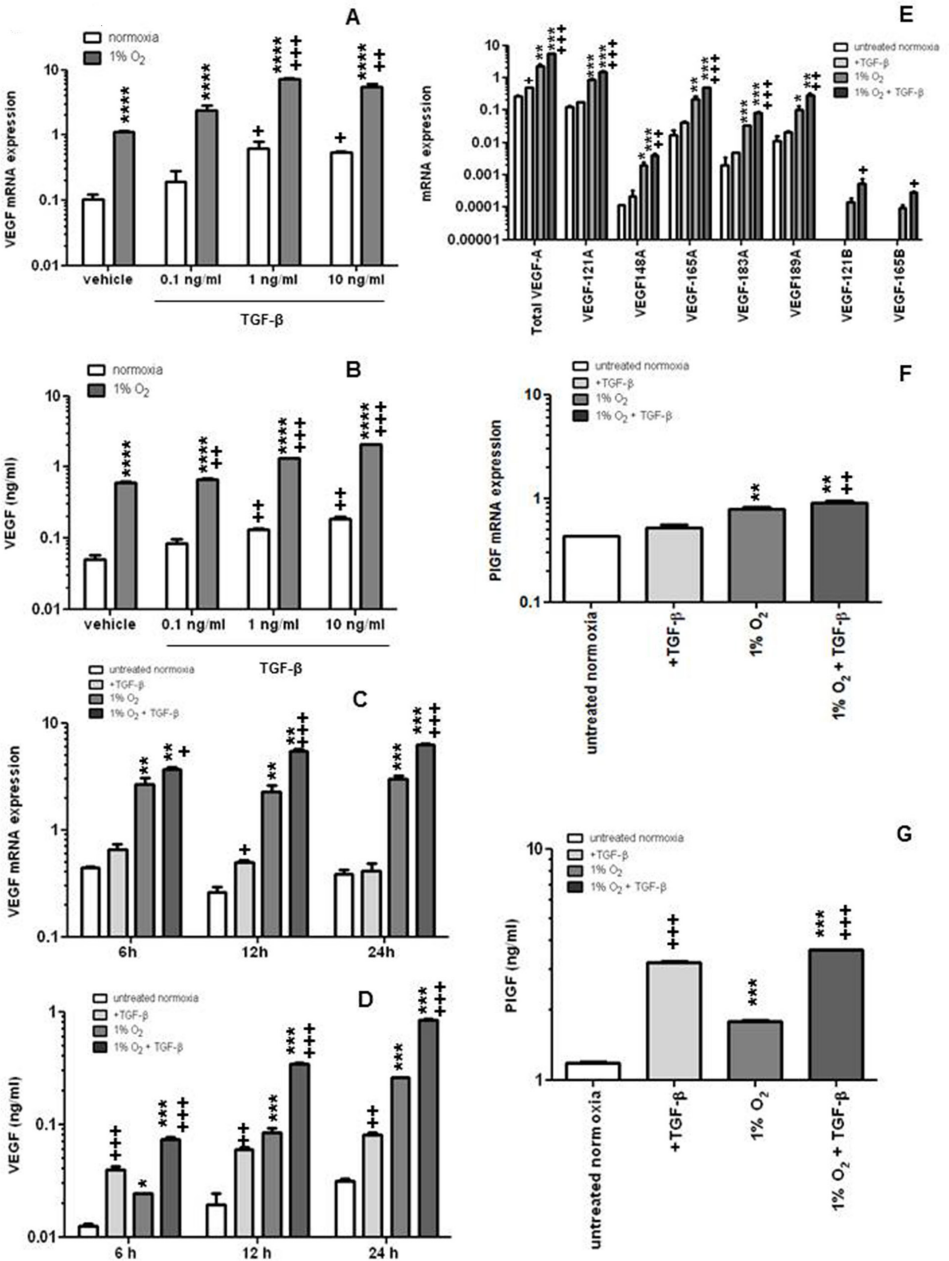
TGF-β induces VEGF and PlGF release by endothelial cells **A, B.** hCMEC were seeded in full medium, serum-starved for 24 h, and then cultured in the absence or presence of TGF-β at the indicated concentrations in normoxia or hypoxia and assessed for VEGF mRNA expression by qRT-PCR at 12 h (A) or VEGF protein concentrations in the supernatant by ELISA at 24 h (B) **C, D.** Similar experiments as in A, B were performed to assess the time dependency of VEGF modulation by TGF-β. **E, F.** The expression of VEGF isoforms (E) or PlGF (F) mRNA was investigated by qRT-PCR (VEGFXXXA, proangiogenic isoforms, VEGFXXXB, weakly angiogenic isoforms). **G.** PlGF concentrations in the supernatant were analyzed by ELISA. Data are expressed as mean and SD (*n* = 3) (*p < 0.05, **p < 0.01, ***p < 0.001, ****p < 0.0001, one-way ANOVA followed by Tukey's post hoc test, hypoxia versus normoxia, ^+^*p* < 0.05, ^++^*p* < 0.01, ^+++^*p* < 0.001, one-way ANOVA followed by Tukey's post hoc test, effect of TGF-β).

We next investigated the effect of recombinant TGF-β on VEGF expression in hCMEC at increasing concentrations for 24 h either at normoxia or under hypoxic conditions (1% O_2_). VEGF mRNA expression and protein release were increased approximately 10-fold when the cells were shifted to hypoxia. Exposure to TGF-β increased VEGF mRNA expression and protein release in a concentration- and time-dependent manner, more significantly even in hypoxia than under normoxic conditions (Figure 2A–2D). The lowest concentration of TGF-β (0.1 ng/ml) did not increase VEGF protein in normoxia whereas the increase was significant in hypoxia. The induction of VEGF mRNA expression was not restricted to a specific VEGF isoform (Figure 2E). Hypoxia increased PlGF mRNA expression, moreover, the combination of hypoxia and TGF-β resulted in a further enhancement compared to hypoxia or TGF-β alone (Figure 2F). PlGF release was increased by TGF-β or hypoxia, and the combination was more effective than either treatment alone (Figure 2G).

### TGF-β-mediated induction of VEGF mRNA and release in hCMEC involves TβRII, ALK-5 and TβRIII

**Figure 3:**
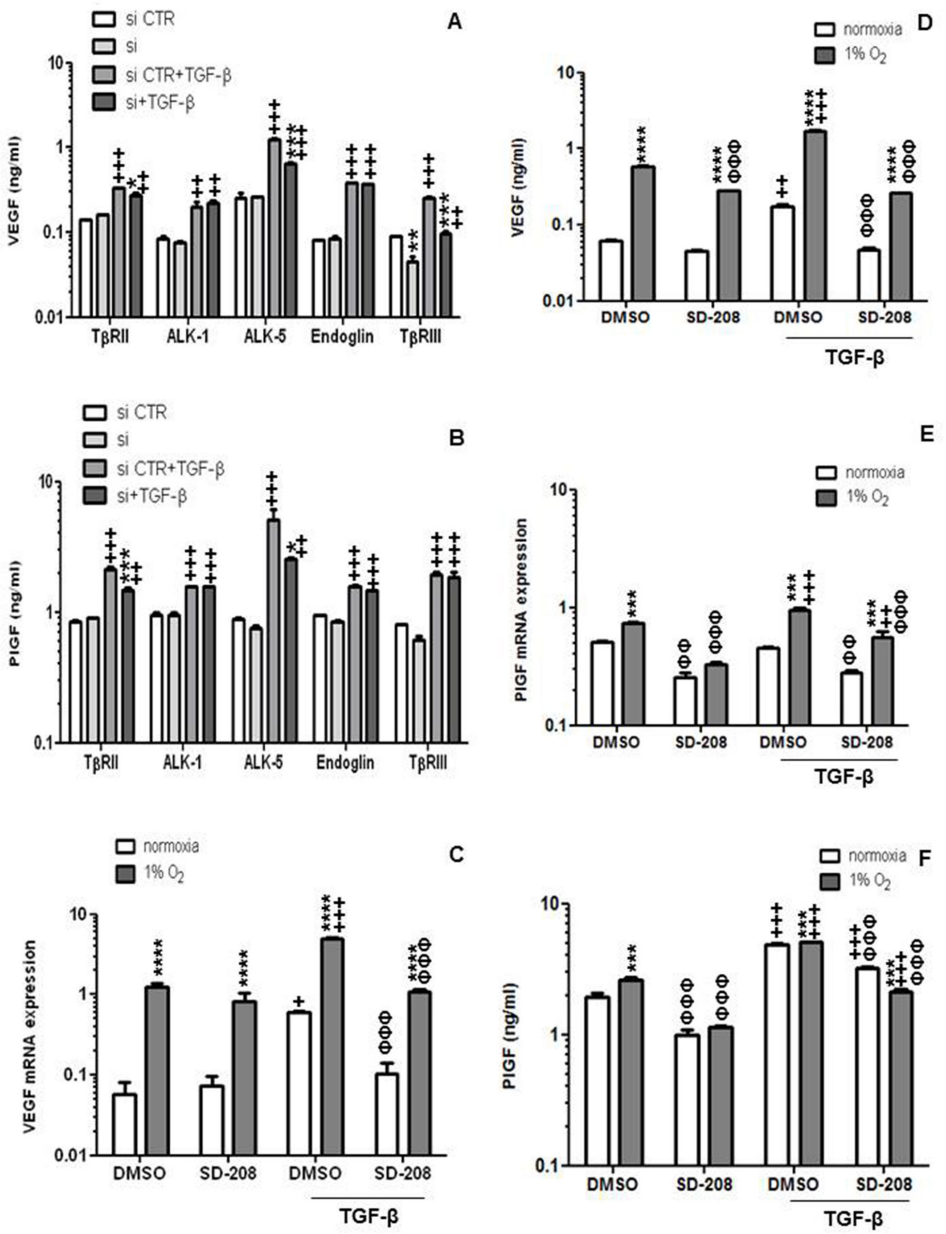
TGF-β-mediated induction of VEGF in endothelial cells involves RII, ALK-5 and RIII **A.** The effect of TGF-β receptor gene silencing on constitutive or TGF-β-evoked VEGF release was assessed by ELISA at 72 h (*p < 0.05, **p < 0.01, ***p < 0.001, one way ANOVA followed by Tukey's post hoc test, effect of specific siRNA, ^+^*p* < 0.05, ^++^*p* < 0.01, ^+++^*p* < 0.001, effect of TGF-β). **B.** The effect of TGF-β receptor gene silencing on constitutive or TGF-β-evoked PlGF release was assessed by ELISA at 72 h (*p < 0.05, **p < 0.01, ***p < 0.001, effect of specific siRNA, ^++^*p* < 0.01, ^+++^*p* < 0.001, effect of TGF-β). **C–F.** The modulation of constitutive or TGF-β-evoked VEGF (C, D) or PlGF (E, F) mRNA expression (C, E) or protein release (D, F) by hypoxia or SD-208 or both was determined by RT-PCR or ELISA. Data are expressed as mean and SD (*n* = 3). (*p < 0.05, *p < 0.01, ***p < 0.001, two-way ANOVA, hypoxia versus normoxia, ^+^*p* < 0.05, ^++^*p* < 0.01, ^+++^*p* < 0.001, one-way ANOVA followed by Tukey's post hoc test, effect of TGF-β, ΦΦΦ *p* < 0.001, one-way ANOVA followed by Tukey's post hoc test, effect of SD-208).

To determine the relative contribution of TGF-β receptors and co-receptors to the TGF-β-mediated increase of VEGF and PlGF in hCMEC, we examined their modulation by gene silencing of TβRII, ALK-1, ALK-5, endoglin or TβRIII. TβRII, ALK-1, ALK-5 and endoglin gene silencing had no effect on constitutive VEGF release whereas silencing of TβRIII reduced VEGF release by 2-fold. In contrast, when VEGF release was induced by exogenous TGF-β, silencing of TβRII, ALK-5 or TβRIII was inhibitory (Figure 3A). Silencing of TGF-β receptors did not modulate constitutive PlGF release, however TβRII or ALK-5 depletion significantly inhibited TGF-β-induced PlGF secretion (Figure 3B).

The important signaling function of ALK-5 was confirmed by pharmacological studies using SD-208 which demonstrated no effect on constitutive VEGF release, but strong activity against TGF-β-evoked VEGF gene transcription and protein release (Figure 3C–3D). This effect was not restricted to specific VEGF mRNA isoforms (Supplementary Figure 3). Under hypoxic conditions, SD-208 even inhibited constitutive VEGF release. The effects of TGF-β and accordingly SD-208 were overall more prominent for PlGF than for VEGF (Figure 3E–3F).

### Transcriptional regulation of TGF-β-evoked VEGF release

To elucidate the transcriptional regulation of VEGF by TGF-β, 2 major transcription factors known to have multiple response elements on the promoter region of VEGF, namely, specificity protein (SP1) and early growth response (EGR)1 were screened for basal mRNA expression in hCMEC, HUVEC and two glioma cell lines, U87MG and LN-18 (Supplementary Figure 4A–4B). All cells expressed SP1 and EGR1. SP1 and EGR1 mRNA expression decreased with TGF-β exposure at 1–6 h in hCMEC, however, protein levels were unaffected (Supplementary Figure 4C). Moreover, the silencing of SP1 did not alter VEGF mRNA expression or protein release (Supplementary Figure 4D). Hence, these data do not support the idea that these transcription factors are involved in the TGF-β-evoked increase of VEGF in hCMEC in normoxia.

### TGF-β induces endothelial mesenchymal transition (EndMT) and TGF-β-stimulated supernatant increases tube formation via VEGFR signaling in hCMEC

**Figure 4:**
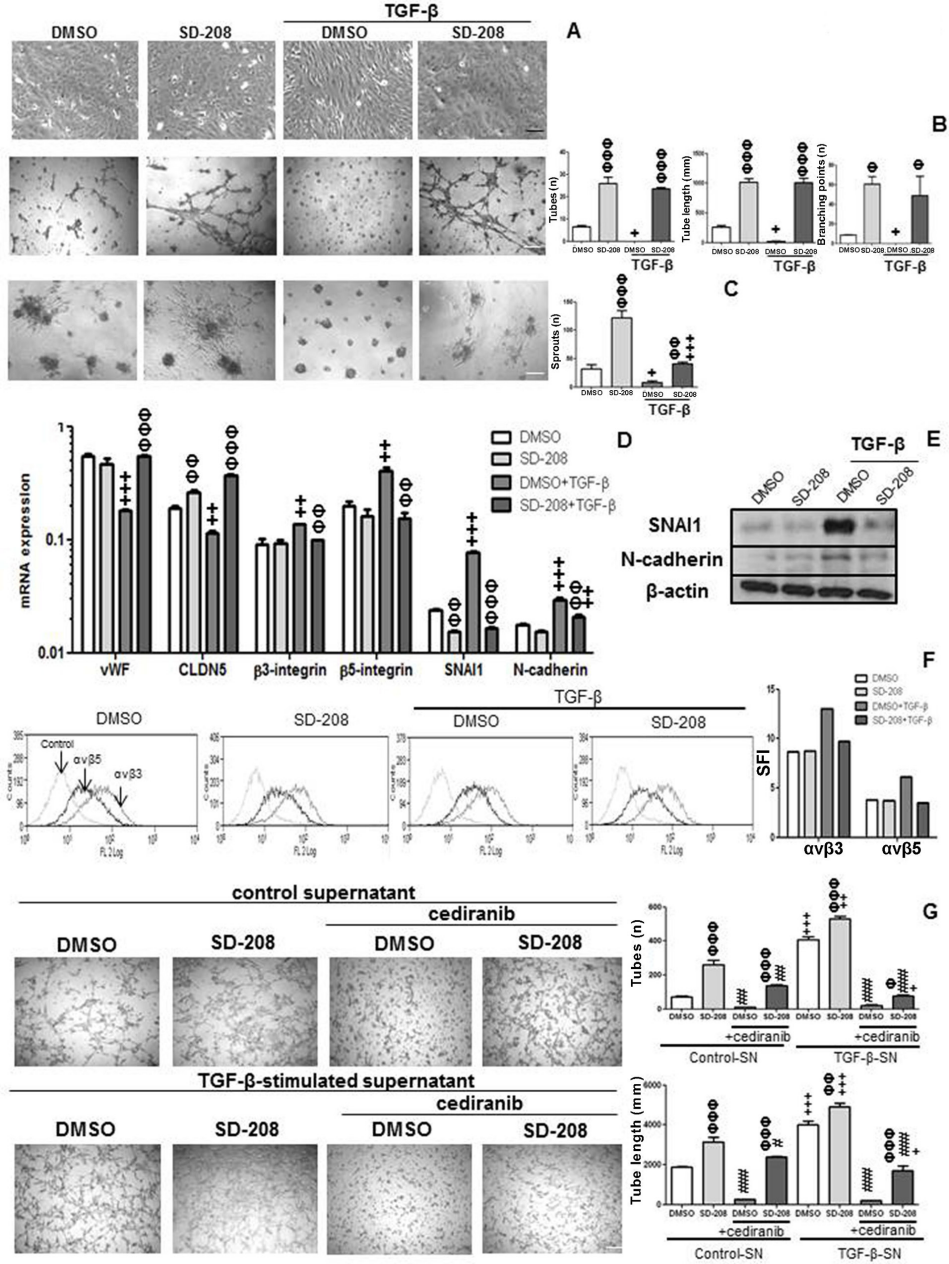
TGF-β induces EndMT in hCMEC and TGF-β-stimulated hCMEC supernatant increases tube formation **A.** hCMEC were seeded in full medium, treated with TGF-β (10 ng/ml) or SD-208 (1 μM) for 96 h, and examined by phase contrast microscopy. **B.** Tube formation was quantified by assessing the number of tubes (n), tube length (mm) and number of branching points (n). **C.** Endothelial cell sprouting was assessed by counting numbers of sprouts (n). **D.** mRNA expression of vWF, CLDN5, β3 and β5 integrins, SNAI1 and N-cadherin was assessed by qRT-PCR under the same conditions as in A, for 72 h. Data are expressed as mean and SD (*n* = 3) (^+^*p* < 0.05, ^++^*p* < 0.01, ^+++^*p* < 0.001, one-way ANOVA followed by Tukey's post hoc test, effect of TGF-β, ΦΦ *p* < 0.01, ΦΦΦ *p* < 0.001, effect of SD-208). **E.** Protein levels of SNAI1 and N-cadherin were assessed by immunoblot under the same conditions as A at 96 h. **F.** Protein levels of αvβ3 and αvβ5 was assessed by flow cytometry under the same conditions as in A, at 96 h. **G.** hCMEC supernatant treated with TGF-β for 96 h was concentrated and added with or without cediranib (1 μM) to hCMEC pretreated with DMSO or 1 μM SD-208 for 1 h. Tube formation was assessed after 12 h, by determining the number of tubes and tube length. Data are expressed as mean and SD (*n* = 3) (ΦΦ *p* < 0.01, ΦΦΦ *p* < 0.001, one-way ANOVA followed by Tukey's post hoc test, effect of SD-208, ^+^*p* < 0.05, ^++^*p* < 0.01, ^+++^*p* < 0.001, effect of TGF-β-stimulated supernatant, #*p* < 0.05, ###*p* < 0.001, effect of cediranib).

We noted that exposure to TGF-β caused changes in the morphology of hCMEC, making the cells more elongated and spindle-like in a SD-208-sensitive, thus presumably ALK-5-dependent manner (Figure 4A). Interestingly, however, SD-208 alone induced tube formation, endothelial cell sprouting and expression of the tight junction marker, CLDN5. Conversely, exogenous TGF-β blocked the formation of tubes and sprouts and decreased vWF and CLDN5 mRNA expression. Pretreatment with SD-208 rescued tube formation, sprouting, vWF and CLDN5 mRNA expression (Figure 4B–4D). Further, TGF-β increased integrin β3 and β5, snail family zinc finger (SNAI) 1 and N-cadherin mRNA expression and protein levels and pretreatment with SD-208 attenuated this increase (Figure 4D–4F). Altogether, the pattern of decreased vWF [4], CLDN5 [5, 6] with a concomitant increase in β3 [7] and β5 integrin expression [8], SNAI1 [5, 9] and N-cadherin [10] are prominent signs of mesenchymal transition. To dissect the apparent opposing pro- and anti-angiogenic effects of TGF-β on hCMEC, we changed the experimental paradigm and isolated the effect of TGF-β-induced VEGF on tube formation and cell sprouting by using supernatants of hCMEC treated with TGF-β for 96 h. These were added to hCMEC that had been pre-treated with vehicle (DMSO) or SD-208. Under these conditions, TGF-β-stimulated supernatant increased tube formation and sprouting. SD-208 super-induced these pro-angiogenic effects of TGF-β-stimulated supernatants, consistent with antagonistic activity of endogenous or supernatant-derived TGF-β and illustrating how TGF-β and VEGF counterbalance the endothelial phenotype. Cediranib, a pan-VEGFR inhibitor, decreased constitutive and TGF-β-stimulated supernatant-induced tube formation in the absence or presence of SD-208 (Figure 4G).

### Endothelial properties of glioblastoma-derived endothelial cells (GMEC)

Finally we wished to confirm the principle observations summarized above in freshly isolated CD31-positive *ex vivo* endothelial cells (ZHE-459, ZHE-464, ZHE-483–2) from glioblastoma tissue. Their endothelial nature was verified by CD31, CD34, and vWF expression and by their tube formation capacity (Supplementary Figure 5A–5B). CD31 mRNA expression in the CD31-positive ZHE-464 cells at P5 was reduced approximately 50 times compared to that on the day of isolation whereas CD31-negative cells did not express CD31 even after 5 passages in endothelial medium, DMEM or neurobasal medium (Supplementary Figure 5C).

### Reduction of VEGF receptor levels in hypoxia in hCMEC and GMEC

**Figure 5:**
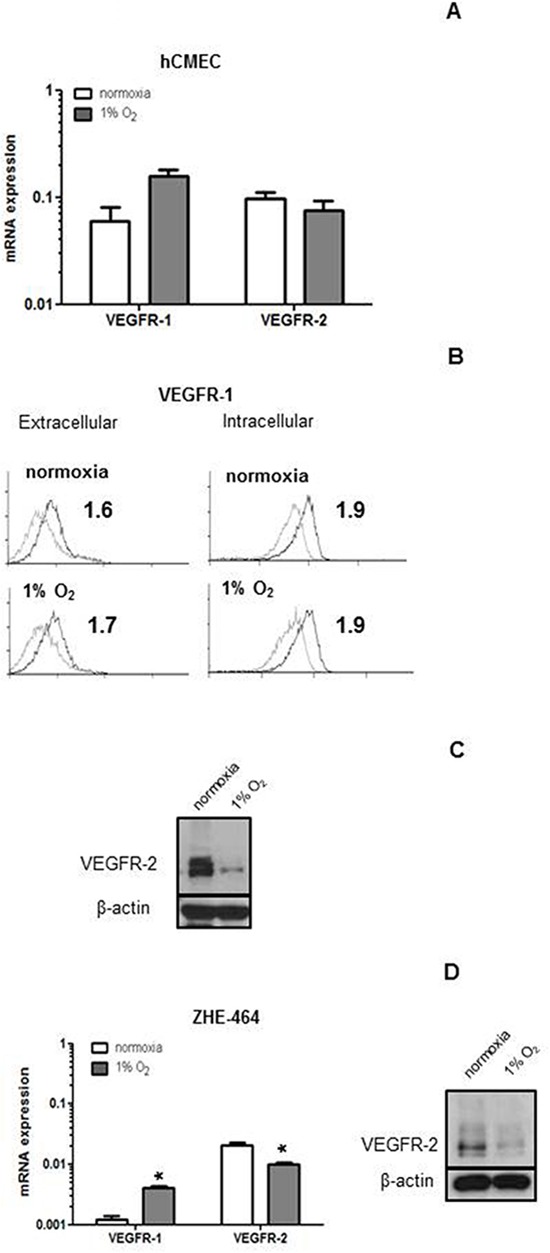
Modulation of VEGFR expression by hypoxia in hCMEC and GMEC **A.** VEGFR-1 or VEGFR-2 mRNA expression were assessed under normoxia or 24 h of hypoxia by qRT-PCR. **B.** Basal levels of extracellular or intracellular VEGFR-1 protein were assessed by flow cytometry under normoxia or hypoxia, SFI are indicated. **C.** VEGFR-2 levels of hCMEC were assessed by immunoblot. **D.** Similar experiments as in A and C were performed in ZHE-464. Data are expressed as mean and SD (*n* = 3). (*, effect of hypoxia, student *t*-test).

The basal expression of VEGFR-1 and VEGFR-2 was determined at mRNA and protein levels at normoxia or after 24 h of hypoxia in hCMEC and GMEC. VEGFR-1 and VEGFR-2 mRNA expression did not change in hypoxia in hCMEC (Figure 5A). VEGFR-1 protein levels at the cell surface and after permeabilization were not altered (Figure 5B). In contrast, there was a strong decrease in VEGFR-2 protein levels under hypoxia as assessed by immunoblot (Figure 5C). In ZHE-464 the mRNA expression of VEGFR-1 increased, whereas VEGFR-2 was reduced at the mRNA and protein levels in hypoxia (Figure 5D), suggesting differential regulation of the VEGF receptors by hypoxia.

### TGF-β control of VEGF release in GMEC

**Figure 6:**
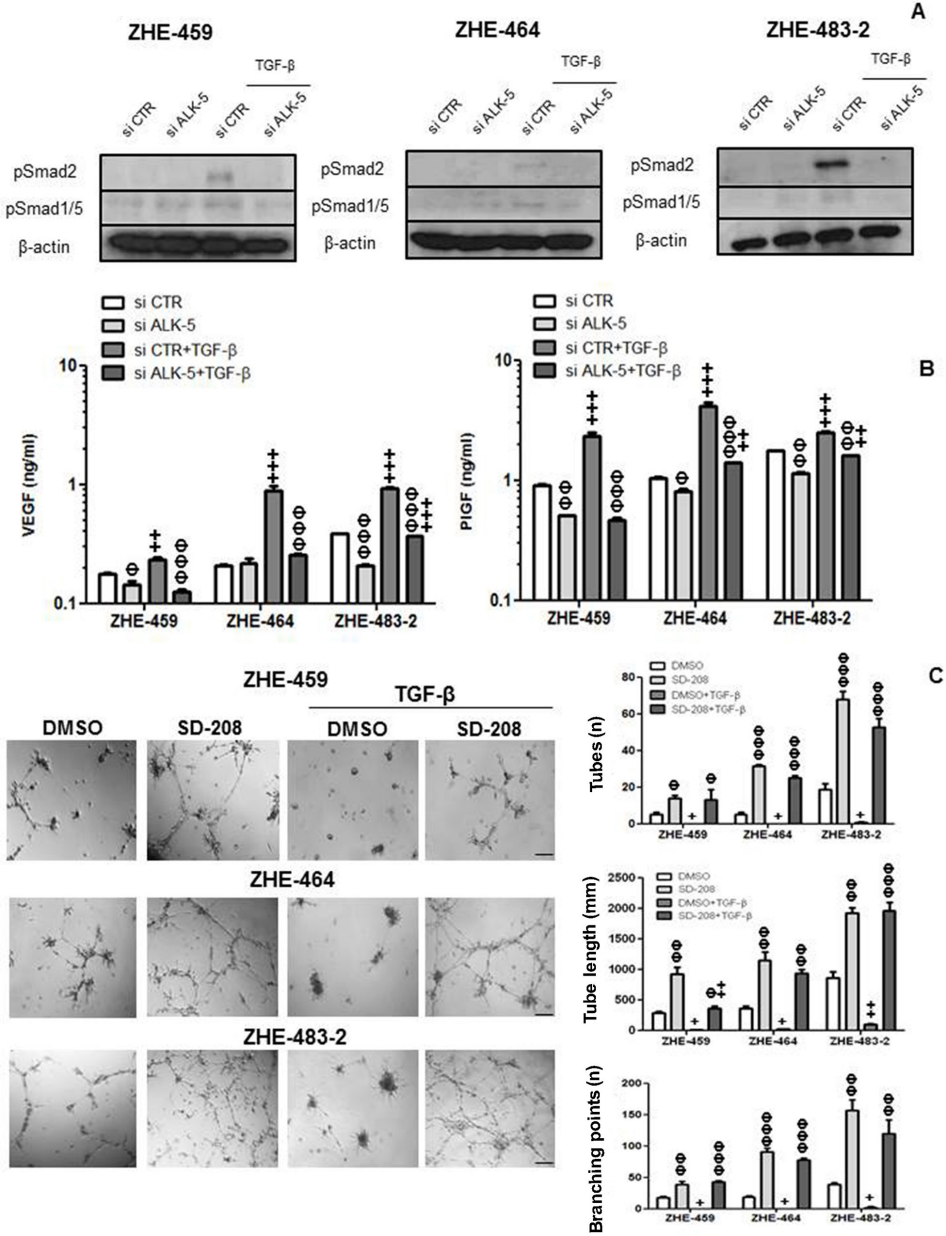
TGF-β control of VEGF release in glioblastoma-derived endothelial cells (GMEC) **A.** The effect of ALK-5 gene silencing on constitutive and TGF-β (10 ng/ml, 72 h)-evoked pSmad2 and pSmad1/5 levels was studied by immunoblot. **B.** The effect of ALK-5 gene silencing on constitutive or TGF-β-evoked VEGF (left) or PlGF (right) release was assessed by ELISA at 72 h in ZHE-459, ZHE-464 and ZHE-483–2. Data are expressed as mean and SD (*n* = 3). (Φ *p* < 0.05, ΦΦ *p* < 0.01, ΦΦΦ *p* < 0.001, one way ANOVA followed by Tukey's post hoc test, effect of si ALK-5, ^+^*p* < 0.05, ^++^*p* < 0.01, ^+++^*p* < 0.001, effect of TGF-β). **C.** ZHE-459, ZHE-464 or ZHE-483–2 cells were seeded in full medium and treated with TGF-β (10 ng/ml) or SD-208 (1 μM) for 96 h. Tube formation was monitored and quantified using number of tubes, tube length and number of branching points as read-outs. Data are expressed as mean and SD (*n* = 3). (Φ *p* < 0.05, ΦΦ *p* < 0.01, ΦΦΦ *p* < 0.001, one way ANOVA followed by Tukey's post hoc test, effect of SD-208, ^+^*p* < 0.05, ^++^*p* < 0.01, ^+++^*p* < 0.001, effect of TGF-β).

Silencing of ALK-5 in the GMEC was confirmed by RT-PCR (Supplementary Figure 5D). Exogenous TGF-β induced pSmad2 and pSmad1/5 in GMEC, and ALK-5 silencing attenuated this effect (Figure 6A). Further, silencing of ALK-5 reduced constitutive VEGF release in ZHE-459 and ZHE-483–2. TGF-β increased VEGF in an ALK-5-dependent fashion in ZHE-459, ZHE-464 and ZHE-483–2 (Figure 6B). Similarly, silencing of ALK-5 reduced constitutive and TGF-β-evoked PlGF release in ZHE-459, ZHE-464 and ZHE-483–2 (Figure 6B).

### TGF-β induces EndMT and TGF-β-stimulated supernatant increases tube formation via VEGFR signaling in GMEC

**Figure 7:**
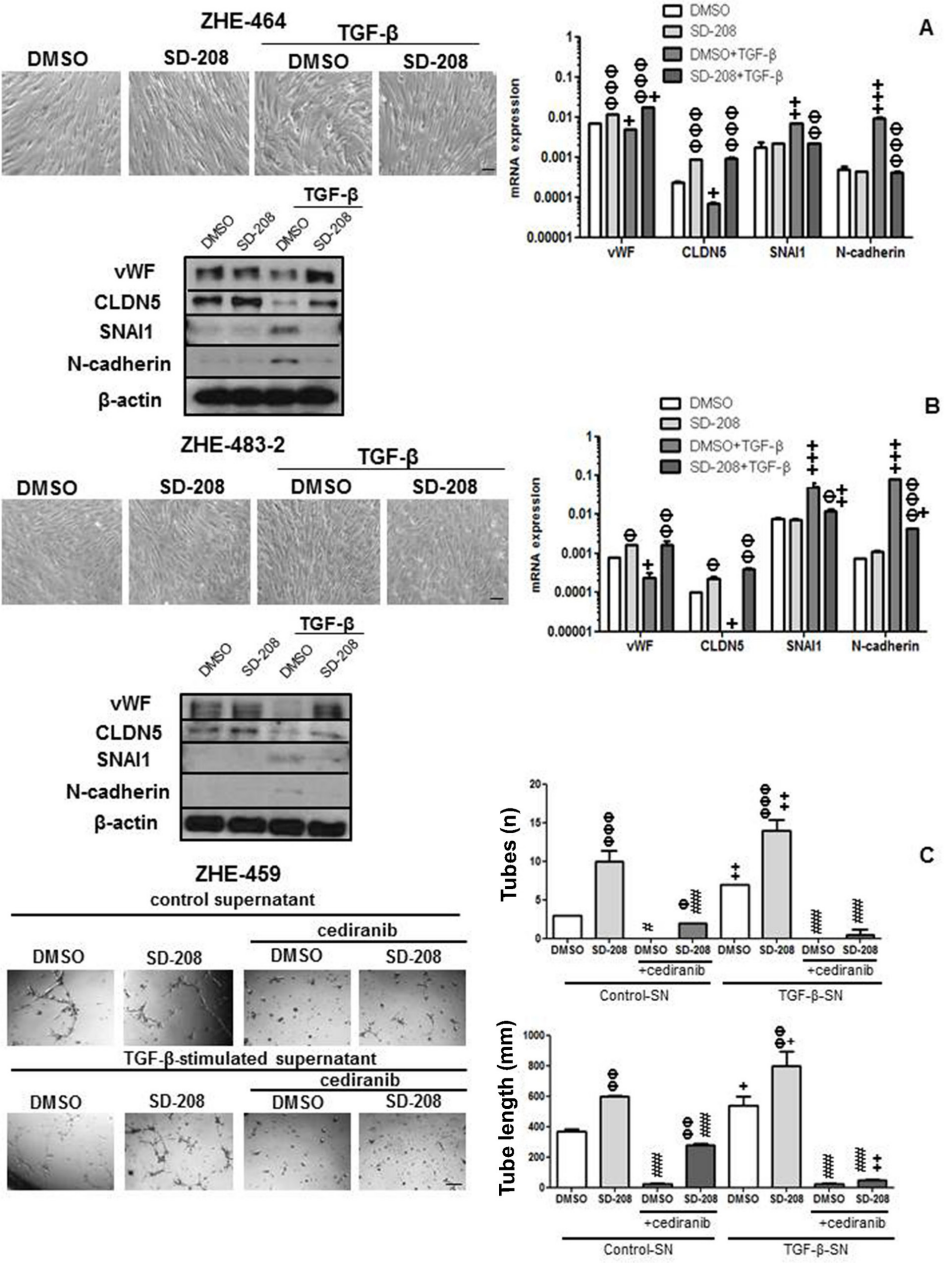
TGF-β induces EndMT and TGF-β-stimulated supernatant increases tube formation in GMEC **A, B.** ZHE-464 (A) or ZHE-483–2 (B) were seeded in full medium and treated with TGF-β (10 ng/ml) or SD-208 (1 μM) for 96 h. Phase contrast images are shown. mRNA expression and protein levels of vWF, CLDN5, SNAI1 and N-cadherin were assessed by qRT-PCR at 72 h and immunoblot at 96 h respectively under the same conditions. Data are expressed as mean and SD (*n* = 3) (Φ *p* < 0.05, ΦΦ *p* < 0.01, ΦΦΦ *p* < 0.001, one way ANOVA followed by Tukey's post hoc test, effect of SD-208, ^+^*p* < 0.05, ^++^*p* < 0.01, ^+++^*p* < 0.001, effect of TGF-β). **C.** ZHE-459 supernatant treated with TGF-β for 96 h was concentrated and added with or without cediranib (1 μM) to ZHE-459 pretreated with DMSO or 1 μM SD-208 for 1 h. Tube formation was assessed after 12 h, by calculating the number of tubes and tube length. Data are expressed as mean and SD (*n* = 3) (ΦΦ *p* < 0.01, ΦΦΦ *p* < 0.001, one-way ANOVA followed by Tukey's post hoc test, effect of SD-208, ^+^*p* < 0.05, ^++^*p* < 0.01, effect of TGF-β-stimulated supernatant, #*p* < 0.05, ##*p* < 0.01, ###*p* < 0.001, effect of cediranib).

Tube formation was enhanced by pharmacological inhibition of ALK-5 in ZHE-459, ZHE-464 and ZHE-483–2. Conversely, TGF-β inhibited tube formation, but pre-treatment with SD-208 rescued tube formation, confirming that TGF-β inhibits angiogenic properties via ALK-5 in GMEC, too (Figure 6C). Further, TGF-β induced EndMT in ZHE-464 and ZHE-483–2. TGF-β reduced the apico-basal polarity of ZHE-464 and created more spindle shaped ZHE-483–2 cells. TGF-β decreased vWF and CLDN5 in ZHE-464 and ZHE-483–2, and concomitantly increased SNAI1 and N-cadherin in an ALK-5-dependent manner, very similar to hCMEC (Figure 7A–7B). Similar to hCMEC, cediranib suppressed the constitutive and TGF-β-stimulated supernatant-induced tube formation in the absence or presence of SD-208 in ZHE-459 (Figure 7C).

## DISCUSSION

Angiogenesis is an important step in the multi-step formation of solid cancers including glioblastoma. It requires a crosstalk between tumor and endothelial cells that is likely to involve much more complex interactions than merely hypoxic tumor cell-derived VEGF acting on endothelial cells. Targeting blood vessel formation in glioblastoma has been the favored therapeutic strategy in clinical drug development in the first decade of this century. Yet, the failure of multiple drugs acting not only on VEGF or its receptors, but also on alternative targets including integrins has imposed a brake on further efforts of targeting angiogenesis and instead called for a better understanding of the processes that govern glioblastoma-associated blood vessel formation [11].

Primary resistance to or escape from anti-angiogenic therapy in glioblastoma is likely to be mediated by compensatory activation of multiple pro-angiogenic pathways and shaped by complex interactions between tumor cells and their microenvironment [12]. VEGF remains to be the major molecule thought to drive endothelial cell growth, migration, vessel dilation and permeability. VEGF, VEGF-B, VEGF-C, VEGF-D and PlGF represent a family of related growth factor molecules which bind to VEGFR1–3 with varying affinity. VEGF binding to VEGFR-2 is critically required for vascular development and sprouting, maturation and differentiation of endothelial cells [13].

TGF-β is a multifunctional cytokine that has been strongly associated with the malignant phenotype of glioblastoma and become an attractive target for pharmacological intervention [14, 15] Here we report that hCMEC express all known TGF-β receptors and coreceptors (Figure 1). Their constitutive VEGF release was largely unaffected by TGF-β receptor gene silencing except for the effect of TβRIII coreceptor silencing (Figure 3A), suggesting that TβRIII exerts activity beyond TGF-β signaling in this paradigm [16]. Exogenous TGF-β induced VEGF mRNA synthesis and protein release in hCMEC in a time- and concentration-dependent manner (Figure 2). The inhibition of VEGF release by ALK-5 gene silencing or pharmacological inhibition using SD-208 led to the identification of ALK-5 as the principal type 1 receptor mediating the VEGF induction by TGF-β (Figure 3), consistent with the observation that ALK-5^−/−^ mouse endothelial cells obtained from E9.5 embryos shows reduced VEGF mRNA expression [17].

TGF-β has been shown to increase PlGF mRNA and protein release in keratinocytes [18] and in retinal epithelium [19]. We show that TGF-β induces PlGF expression and release in endothelial cells in normoxic (Figure 2, Figure 3 and Figure 6) and hypoxic conditions (Figure 3). We further show that ALK5 mediates the effects of TGF-β on PlGF and that hypoxia may super-induce the PlGF system in the context of glioblastoma.

We observed that VEGFR-2 protein levels in hCMEC and GMEC decreased under hypoxia (Figure 5). Up-regulation of VEGFR-1 and down-regulation of VEGFR-2 at the mRNA and protein levels have been reported previously in HUVEC exposed to hypoxia [20, 21]. The promoter regions of VEGFR-1 that have HIF response elements (HRE) were identified whereas no obvious HRE on the VEGFR-2 promoter region was detected. It was speculated that the regulation of VEGFR-2 in hypoxia was mediated by an unindentified paracrine soluble factor [20] or through post-transcriptional mechanisms [21]. In response to VEGF-A, hypoxic human dermal microvascular endothelial cells (HDMEC) displayed a reduction in VEGFR-2 phosphorylation compared with normoxia, indicating reduced VEGF-A-induced receptor activation in hypoxia [22].

We find that TGF-β induces EndMT in hCMEC and GMEC as determined by a decrease in the expression of the endothelial markers vWF and CLDN5, inhibition of tube formation and an increase in the expression of the mesenchymal markers SNAI1 and N-cadherin in an ALK-5-dependent manner (Figure 4 and Figure 7). TGF-β-induced mesenchymal transition of mouse pancreatic microvascular endothelial cells required Smad-dependent cooperative activation of Rho signals and MRTF-A [23]. TGF-β promoted SNAI1-mediated EndMT through convergence of Smad-dependent and Smad-independent signalling in human cutaneous microvascular endothelial cells [24]. Sustained elevation of SNAI1 has been suggested to promote a glial-mesenchymal transition in gliomas after radiation [25].

**Figure 8:**
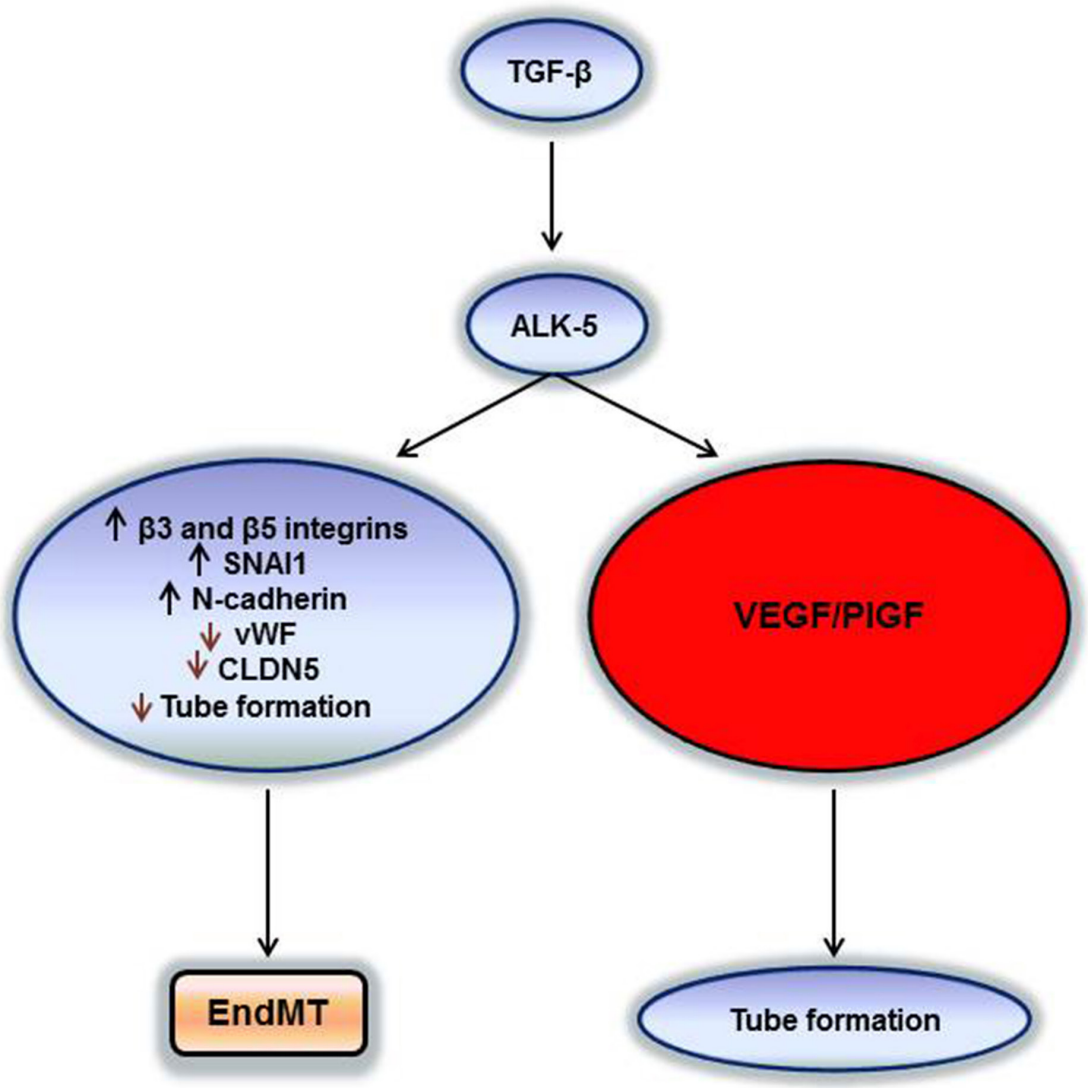
Two pathways of TGF-β/ALK-5 signalling in hCMEC and GMEC TGF-β can increase VEGF/PlGF secretion via the ALK-5 pathway and create a pro-angiogenic milieu for the tumor or induce EndMT by decreasing endothelial properties and increasing the mesenchymal signatures for the tumor to become potentially more invasive.

VEGF and TGF-β signalling may induce a plethora of gene expression changes observed in glioblastoma vessels *in vivo* [26]. The dual effects of TGF-β on biological aspects of angiogenesis characterized here (Figure 8) makes the consequences of therapeutic targeting of either pathway difficult to predict. It must be acknowledged that therapeutic targeting of VEGF receptors by cediranib has failed in glioblastoma [27] whereas bevacizumab, while convincingly delaying disease progression, also did not impact overall survival [28, 29]. Yet, it has remained uncertain to what extent these drugs truly reached their desired site of action which must include the tumor microenvironment. Blood brain barrier permeability may have limited the efficacy of cediranib and bevacizumab would *a priori* only be able to scavenge humoral VEGF, but probably not be able to prevent autocrine signaling that is likely to be relevant in glioblastoma, too [30]. Accordingly, control of VEGF synthesis and release is still a valid target in glioblastoma.

## MATERIALS AND METHODS

### Reagents

Recombinant human TGF-β_2_ was purchased from R&D Systems (Minneapolis, MN, USA). All experiments reported herein involving exogenous supplementation were performed with human TGF-β_2_. Selected experiments were confirmed using human TGF-β_1_ (data not shown). SD-208 [14], a TGF-βRI kinase inhibitor was provided by Scios (Fremont, CA, USA). Cediranib (AZD2171) was purchased from Biovision (Milpitas, CA, USA).

### Cell culture

Human cerebral microvascular endothelial cells (hCMEC/D3), kindly provided by P.C. Couraud (Paris, France), human glioblastoma-derived endothelial cells ZHE-459, ZHE-464 and ZHE-483–2 and human umbilical vein endothelial cells (HUVEC) were cultured in Lonza EBM^™^-2 medium (CC-3156, Lonza, Walkersville, MD, USA) with supplements (EGM-2-CC4176) that include 0.5 ml human recombinant epidermal growth factor (hEGF, CC-4317A), 0.2 ml hydrocortisone (CC-4112A), 2 ml human fibroblast growth factor-B (hFGF, CC-4113A), 0.5 ml VEGF (CC-4114A), 0.5 ml recombinant long-insulin like growth factor (R^3^-IGF-1, CC-4115A), 0.5 ml ascorbic acid (CC-4116A), 0.5 ml heparin (CC-4396A), 0.5 ml gentamicin sulphate amphotericin B (GA-1000, CC-4381A), 10 ml (2%) fetal bovine serum (FBS, CC-4101A) and 5 ml (1 M) HEPES (Gibco, GrandIsland, NY, USA) and 5 ml CD lipid concentrate (Gibco). hCMEC/D3 were cultured from P30-P37 on Corning^®^ BioCoat™ collagen type I cellware (Corning) and used for experiments. HUVEC, ZHE-459, ZHE-464 and ZHE-483–2 cells were cultured from P2-P7 on the same dishes as hCMEC/D3. All cells were grown in a humidified 37°C incubator with 5% CO_2_. All starvation experiments for endothelial cells were performed with plain Lonza EBM^™^-2 medium without any supplements. LN-308, a longterm adherent glioma cell line, was cultured in Dulbecco's modified eagle's medium (DMEM) containing 10% fetal calf serum (FCS) and 1% glutamine (Gibco). For all experiments LN-308 cells were starved for 24 h in DMEM containing 1% glutamine.

### Isolation of human glioblastoma-derived endothelial cells

The net weight of the glioma tissue was measured followed by dissociation with 10 mg/ml collagenase/dispase (11097113001, Roche, Basel, Switzerland) and rotation with gentleMACS C-tubes (130–093-237, Miltenyi biotech, Cologne, Germany). Erythrocytes were lysed by resuspending and incubating the cells in a 3:1 ratio of water:PBS. Cells were then counted and 20 μl of FcR blocking reagent and 20 μl of CD31 microbeads (130–091-935, Miltenyi biotech) were added per 10^7^ cells. After 15 min rotating incubation at 4°C, the cells were passed through a 40 μm cell strainer to remove cell aggregates. Cells were then passed through an LS column placed in a magnetic field of a MACS separator. The cells that pass through (CD31-negative population) were again passed through another LS column to get a purer CD31-negative population. These cells were divided into 3 fractions and grown in Neurobasal medium (NBM) supplemented with B-27 (20 μl/ml) and Glutamax (10 μl/ml) (Invitrogen, Carlsbad, CA, USA), FGF and EGF (20 ng/ml each; Peprotech, Rocky Hill, PA, USA), DMEM supplemented with 10% FCS and 1% L-glutamine (Gibco) or endothelial medium (EM). The CD31-positive cells which remained in the column were forcefully washed out and cultured in collagen type I-coated dishes until confluency and passaged once more before use. For ZHE-483–2, CD45-positive cells were depleted using LD columns (130–042-901, Miltenyi biotech), and then from the CD45-negative population, CD31-positive cells were isolated using the above procedure.

### Real-time (RT) PCR

Total RNA was prepared with the NucleoSpin System (Macherey-Nagel, Düren, Germany) and cDNA was transcribed using Superscript II reverse transcriptase (Bio-rad, Munich, Germany). For real-time PCR, cDNA amplification was monitored using SYBR Green chemistry on the 7300 Real time PCR System (Applied Biosystems, Zug, Switzerland). The conditions for these PCR reactions were: 40 cycles, 95°C/15 sec, 60°C/1 min, using the primers specified in Supplementary Table 1. Arf1 transcript levels were used as a reference for relative quantification of mRNA expression levels using the ΔCT method because they were observed to be stable within each cell line under the experimental conditions reported here.

### Immunoblot

RIPA buffer (pH 7.8) consisting of 1% V/V NP-40, 5% sodium deoxycholate (0.5% V/V) (Sigma Aldrich, Buchs, Switzerland), 1 M Tris/HCl pH 8.0 (Merck KGaA, Darmstadt, Germany), 5 M NaCl (Merck KGaA), 0.5 M EDTA, pH 8.0 (Sigma) and MilliQ H_2_O was prepared, sterile-filtered and stored at 4°C. Phosphatase inhibitor cocktail (Sigma), protease inhibitor cocktail sets III and IV (Sigma), 200 mM sodium orthovanadate (pH 10) and 0.5 M sodium fluoride were added freshly to the RIPA buffer before lysing the cells. After this, protein levels were determined using a Bradford-based protein assay (Biorad). Denatured whole protein lysates (40 μg/lane) were separated on 10–15% acrylamide gels. After transfer to nitrocellulose (Biorad), blots were blocked in TBST containing 5% skim milk and incubated overnight at 4°C with primary antibodies. Rabbit anti-human TβRII (1:1000) ((C-16)-R, sc220-R, Santa Cruz Biotechnology, Santa Cruz, CA, USA), rabbit anti-CD105 (1:3000) (ab137389, abcam, Cambridge, UK), goat anti-TβRIII (1:2000) (AF-242-PB, R&D Systems), rabbit anti-VEGFR-2 (1:1000) (Cell Signaling, Danvers, MA), rabbit anti-pSmad2 (1:1000) (3108S, Cell Signaling), rabbit anti- Smad2 (1:1000) (3122S, Cell Signaling), rabbit anti-Smad1 (1:1000) (9743S, Cell Signaling), rabbit anti-Smad5 (1:1000) (9517S, Cell Signaling), rabbit anti-pSmad1/5 (1:1000) (9516S, Cell Signaling), rabbit anti-vWF (1:1000) (sc-14014, Santa Cruz Biotechnology), rabbit anti-CLDN5 (1:1000) (ABT45, Millipore, Billerica, MA, USA), rabbit anti-Snail (1:1000) (3879S, Cell Signaling), mouse anti-N-cadherin (1:2500) (05–915, Millipore), rabbit anti-SP1 (1:1000) (07–645, Millipore), rabbit anti-EGR-1 (1:1000) (sc-110, Santa Cruz Biotechnology) or goat anti-actin (1:500) (sc 1616, Santa Cruz Biotechnology) were used as primary antibodies. The membranes were then washed in TBST and incubated for 1 h at room temperature with HRP-coupled goat anti-rabbit (1:5000) (Santa Cruz Biotechnology) or donkey anti-goat (1:5000) (Santa Cruz Biotechnology) secondary antibodies. Protein bands were visualized by enhanced chemo luminescence (Pierce/Thermo Fisher, Madison, WI, USA).

### ELISA

hCMEC or GMEC were seeded at 2.6 × 10^4^ cells/cm^2^. Indicated treatments were performed in basal medium without serum and supplements for indicated time periods. Supernatants were harvested and cellular debris was removed by centrifugation 1200 rpm for 10 min. Supernatants were then concentrated by centrifugation at 4000 g for 30 min using the Amicon^®^ Ultra 3K device 3,000 NMWL (Millipore). Protein concentrations in supernatants were measured by BCA assay (Pierce Biotechnology, Rockford, IL, USA) and equal amounts of protein were used for VEGF-A ELISA (BMS277/2TEN, eBioscience, Vienna, Austria) and PlGF ELISA (RAB0404, Sigma). Pure serum-free medium without cells was used as a negative control for all ELISA.

### RNA interference

To silence TβRII, ALK-1, ALK-5, endoglin or SP1, hCMEC cells were transiently transfected using Metafectene Pro transfection reagent (Biontex, San Diego, CA, USA) and siRNA pools (100 nM), containing four selected siRNA duplexes, each with a modification pattern that eliminates off-target effects caused by both strands (ON-TARGETplus, SMARTpool, (TβRII-L-003930–00, ALK-1- L-005302–02, ALK-5- L-003929–00, endoglin-L-011026–00, SP1–026959-00) Dharmacon, Lafayette, CO, SA, USA). ON-TARGETplus non-targeting pool siRNA (Dharmacon) was used as a negative control. For TβRIII, three selected siRNA duplexes (S100049595, S100049602, S10049609) were used along with a control non-targeting siRNA pool from Qiagen (Hilden, Germany) with the same transfection reagents and siRNA concentration as used for the silencing of other receptors. Gene silencing was verified by qPCR and immunoblot as indicated.

### Angiogenesis-related *in vitro* assays

hCMEC were cultured in full medium with TGF-β or SD-208 or both for 96 h before being detached using accutase (PAA Laboratories, Cölbe, Germany) and transferred to growth factor-reduced matrigel (356230, BD Biosciences, Franklin Lakes, NJ, USA) in 96 well plates at 15,000 cells/50 μl. Tube formation was assessed at 4, 12 and 24 h and photographs were taken using a Carl Zeiss microscope (Axiovert 100, Göttingen, Germany). Tube length (mm), number of tubes (n) and number of branching points (n) were calculated as average of 3 images per condition from independent experiments using the Adobe Acrobat X Pro (Adobe, Ireland).

For the sprouting assay, the conditions were same as for tube formation except that 2, 000 cells/50 μl were transferred to 96 well plates containing growth factor-reduced matrigel and were left in the matrigel for 7 days. Then the number of sprouts formed by the cells was counted for 3 images per condition from independent experiments using ImageJ 1.40g software (NIH).

### Flow cytometry, cell cycle, and viability assays

hCMEC were cultured in full medium followed by serum starvation for 24 h in normoxia or hypoxia. The cells were then detached using accutase (PAA Laboratories), stained with goat anti-VEGFR-1/Flt-1 (AF321, R&D Systems), mouse anti-αvβ3 (clone LM609, MAB1976) and mouse anti-αvβ5 (clone P1F6, MAB1961) (Merck, Darmstadt, Germany) or appropriate isotype controls: goat IgG, (sc-2028, Santa Cruz Biotechnology) or mouse IgG_2a_ ĸ (550339, BD Biosciences). After 2 washing steps with PBS containing 0.5% bovine serum albumin (BSA), cells were incubated at 4°C with the following secondary antibodies: anti-human alexa fluor 488 antibody (Invitrogen), anti-goat IgG-FITC (F2016, Sigma Aldrich) or anti-mouse IgG1-PE (RMG1–1, Biolegend, London, UK). For the detection of endoglin, mouse PE anti-human CD105 antibody (323205, Biolegend) or the isotype control PE mouse IgG1K (400113, Biolegend) and for TβRIII, goat PE anti-human TβRIII (FAB242P, R&D Systems) or the isotype control PE goat IgG (403004, Biolegend) were used. Subsequently, the cells were washed twice with PBS containing 0.5% BSA and subjected to flow cytometric analysis. A Cyan^®^ Dako flow cytometer was used and data were analyzed via Summit^®^ software version 4.3 (Beckmann Coulter, Krefeld, Germany). Signal intensity was calculated as the ratio of the mean fluorescence of the specific antibody and the isotype control antibody (specific fluorescence index, SFI). A SFI of 1.3 was arbitrarily defined as a significant surface expression. For some analyses, cells were permeabilized by Fix/Perm Buffer Set (Biolegend).

For analysis of cell death, cells were grown in 6-well plates and treated with TGF-β (10 ng/ml) for 72 h. Cells were then harvested, annexin (Anx) V-fluorescein isothiocyanate (1:100) and propidium iodide (PI; 50 μg/ml) were added, and fluorescence in a total of 10,000 events (cells) per condition was recorded in the flow cytometer. Annexin V- or PI-positive cells were counted as dead cells, the remaining cells were designated the surviving cell fraction. Loss of viability was also confirmed by trypan blue dye exclusion [31].

### Statistical analysis

Data are commonly derived from experiments performed at least twice in duplicates with similar results. Quantitative data are represented as mean ± standard deviation (SD) from the mean. Statistical significance was assessed using the student *t*-test, one-way ANOVA with Tukey's post hoc test or a two-way ANOVA wherever applicable. A *p* value below 0.05 was considered significant. All statistical analyses were performed using Prism 5 (GraphPad Software, La Jolla, CA, USA) at *p* < 0.05, *p* < 0.01, *p* < 0.001 or *p* < 0.0001.

## SUPPLEMENTARY FIGURES AND TABLE


