# Supplementary material for: Modulation of cerebral endothelial cell function by TGF-β in glioblastoma: VEGF-dependent angiogenesis versus endothelial mesenchymal transition
Source: Oncotarget. 2015 Jun 15;6(26):22480–95. doi: 10.18632/oncotarget.4310 (PMC4673177; doi:10.18632/oncotarget.4310)
Supplement: Supplementary file 1 [file oncotarget-06-22480-s001.pdf]

SUPPLEMENTARY FIGURES AND TABLE

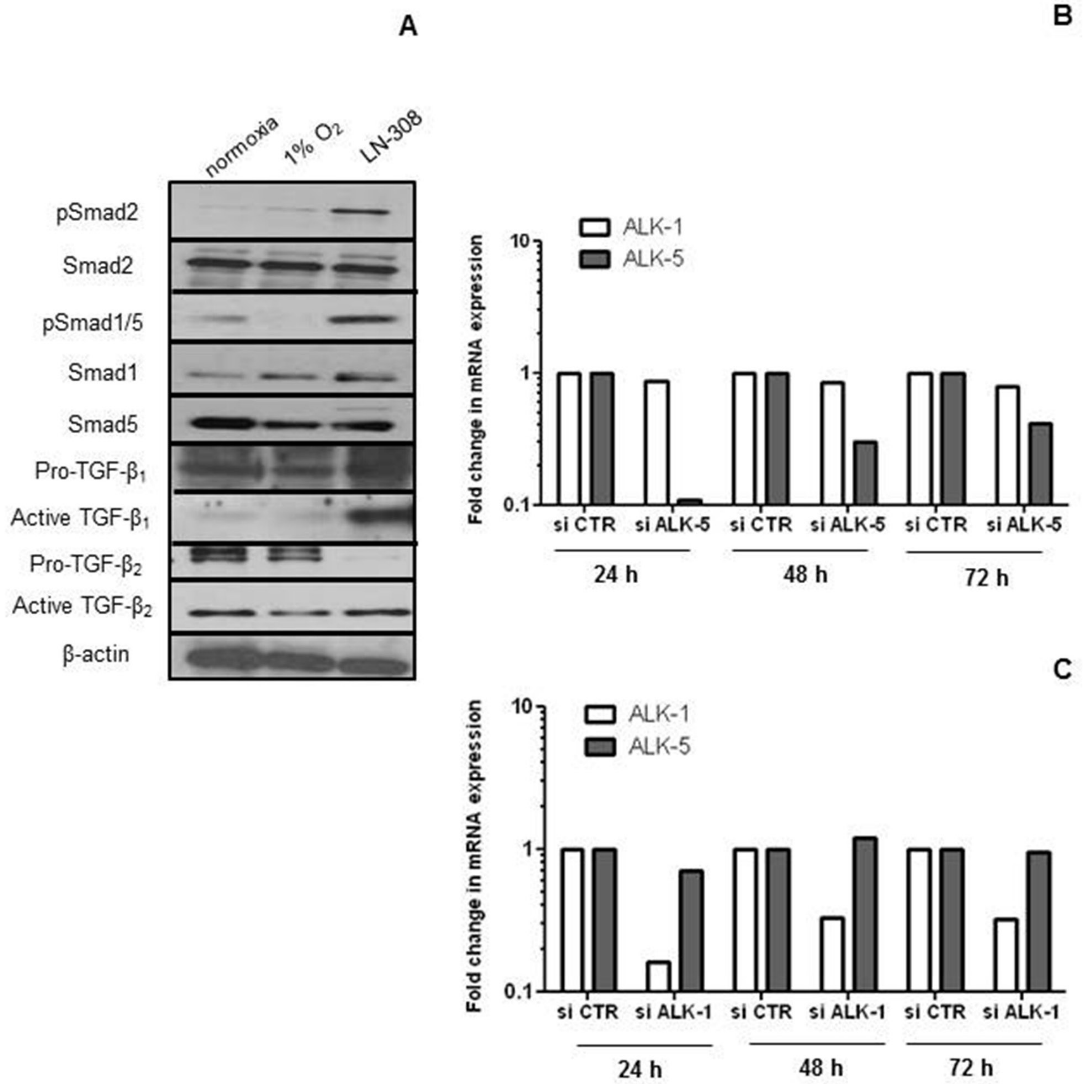

Supplementary Figure 1: Modulation of TGF-β and TGF-β signaling by hypoxia in hCMEC. A. Basal levels of pSmad2, Smad2, pSmad1/5, Smad1 and Smad5 in cell lysates and basal levels of secreted TGF-$\beta_{1,2}$ in the supernatant were detected by immunoblot in normoxia or after 24 h hypoxia. LN-308 glioma cell lysates and supernatants were used as a positive control. **B–C.** hCMEC were seeded in full medium, exposed to siRNA sequences against ALK-1 or ALK-5 for 12 h in serum-free medium, and were then assessed for the specificity of gene silencing by qRT-PCR in a time-dependent manner from 24 to 72 h.

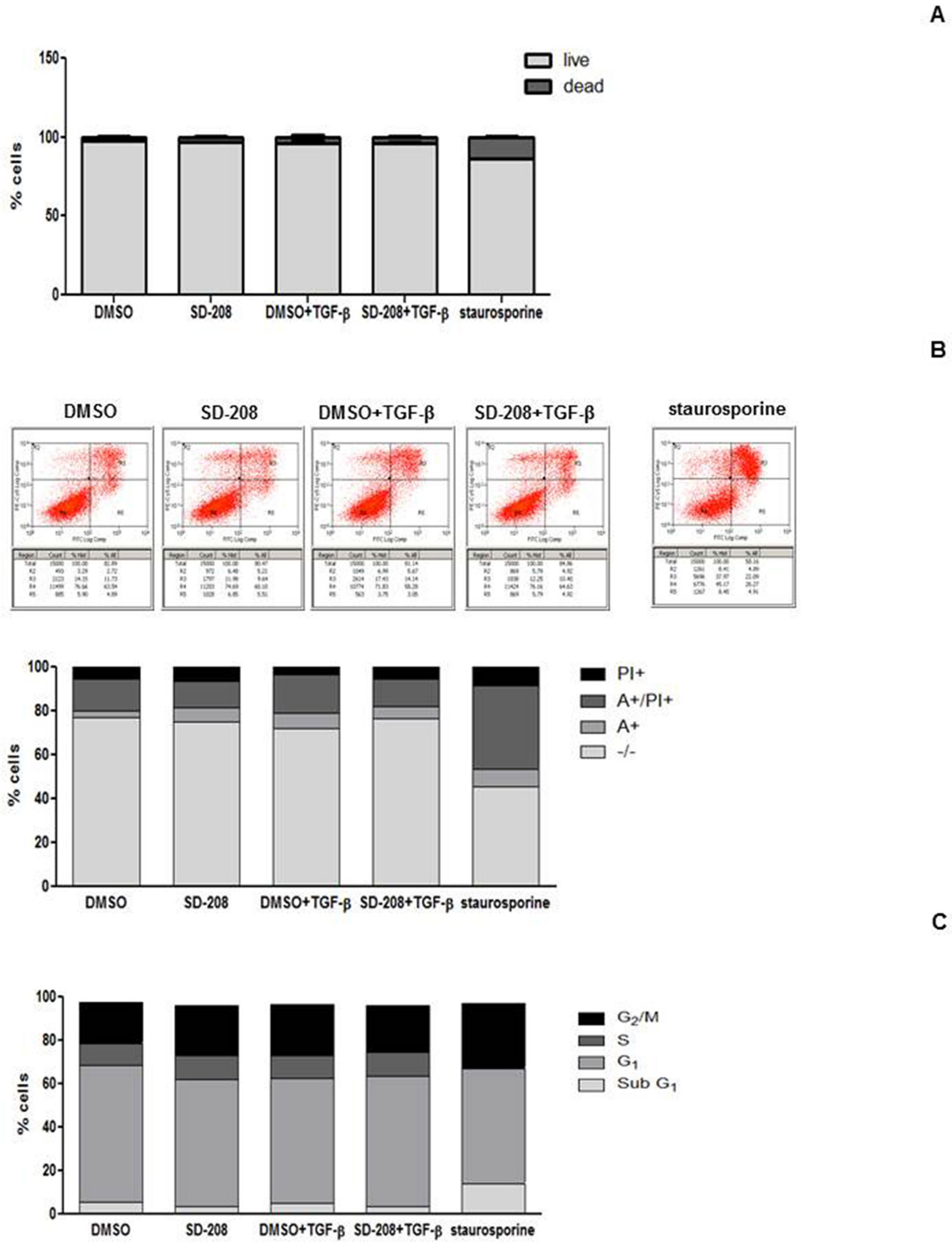

Supplementary Figure 2: Effects of TGF-β on hCMEC proliferation and viability. A. hCMEC were seeded in full medium and treated with TGF-β (10 ng/ml) or 1 µM SD-208 for 96 h, followed by trypan blue staining and counting of viable and dead cells. The floating cells were taken into account. **B.** Cell death was analysed using Annexin V/PI staining. **C.** Cell cycle analysis was performed using PI staining. Cells treated with 2 µM staurosporine for 12 h were used as a positive control.

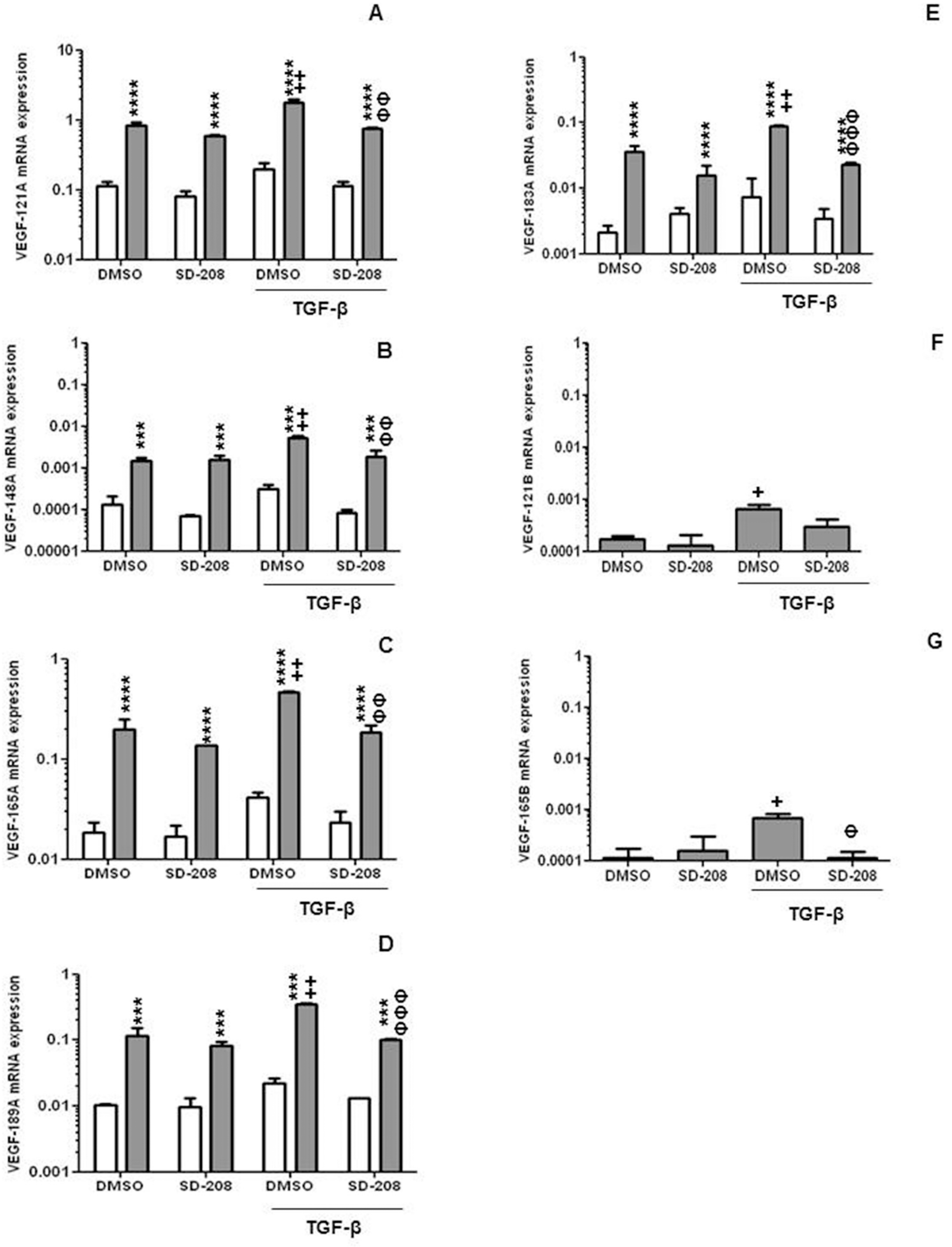

Supplementary Figure 3: VEGF mRNA isoform expression: modulation by hypoxia, TGF-β or SD-208. A–G. hCMEC were seeded in full medium, serum-starved for 24 h, and then cultured in the absence or presence of TGF-β (10 ng/ml) or SD-208 (1 µM) or both in normoxia or hypoxia for 12 h and assessed for VEGF isoform expression by qRT-PCR. Data are expressed as mean and SD ($n = 3$) (***$p < 0.001$, ****$p < 0.0001$, two-way ANOVA, hypoxia vs normoxia, Φ $p < 0.05$, ΦΦ $p < 0.01$, ΦΦΦ $p < 0.001$, one-way ANOVA followed by Tukey's post hoc test, effect of SD-208, $^{+}p < 0.05$, $^{++}p < 0.01$, one-way ANOVA followed by Tukey's post hoc test, effect of TGF-β).

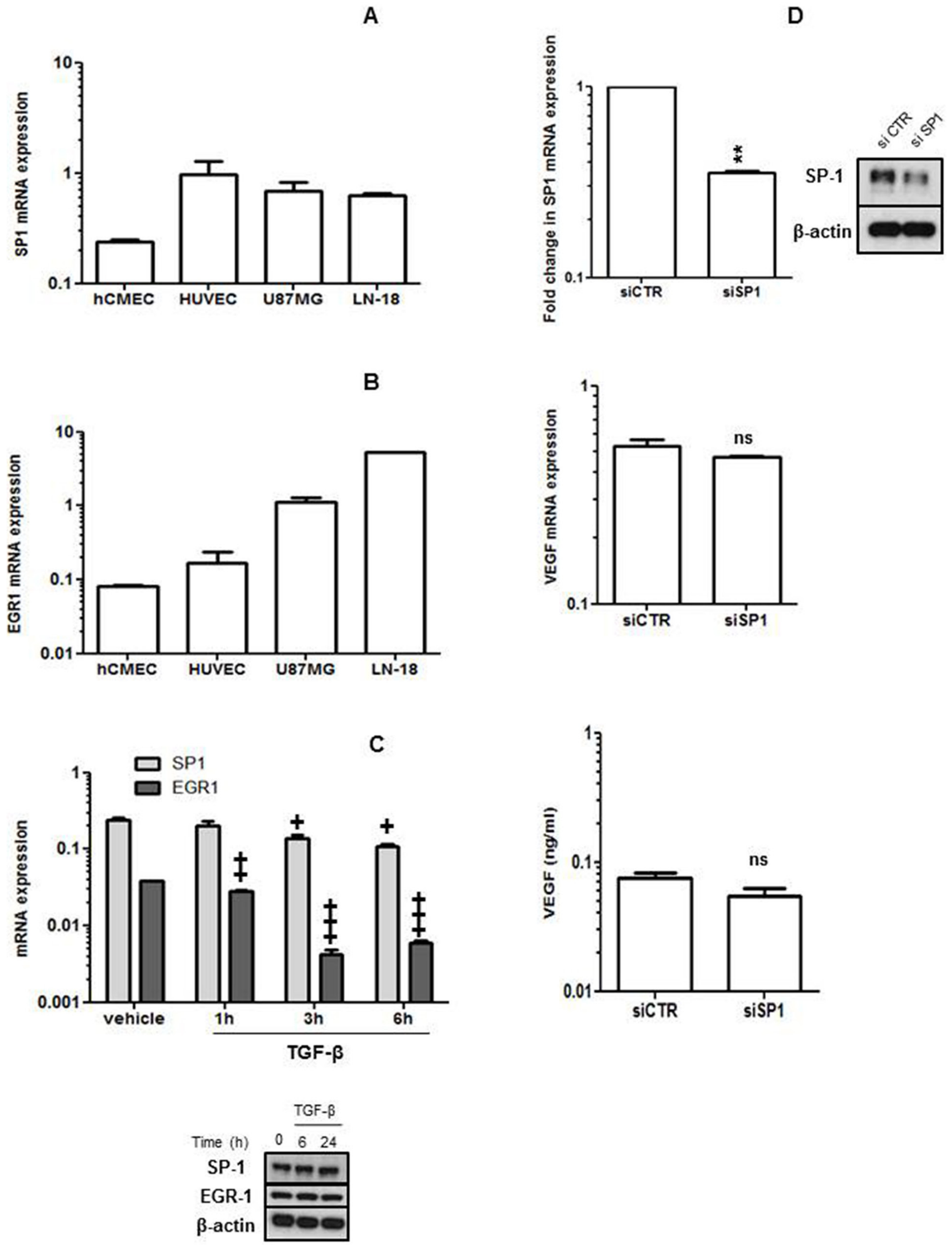

Supplementary Figure 4: TGF-β regulation of VEGF mRNA expression: transcription factor profiling. A, B. Cells were seeded in full medium, serum-starved for 24 h, followed by analysis of the basal mRNA expression of SP1 (A) or EGR1 (B) by qRT-PCR in hCMEC, HUVEC, U87MG or LN-18 cells. **C.** hCMEC were cultured with or without TGF-β (10 ng/ml) at 1, 3 or 6 h and assessed for SP1 and EGR1 mRNA expression by qRT-PCR ($^{+}p < 0.05$, $^{++}p < 0.01$, $^{+++}p < 0.001$ one-way ANOVA followed by Tukey's post hoc test, effect of TGF-β). **D.** Silencing of SP1 was confirmed at mRNA and protein levels. VEGF mRNA expression and protein release were assessed after the silencing of SP1. Data are expressed as mean and SD ($n = 3$). (**, effect of siSP1, student *t*-test); ns (not significant).

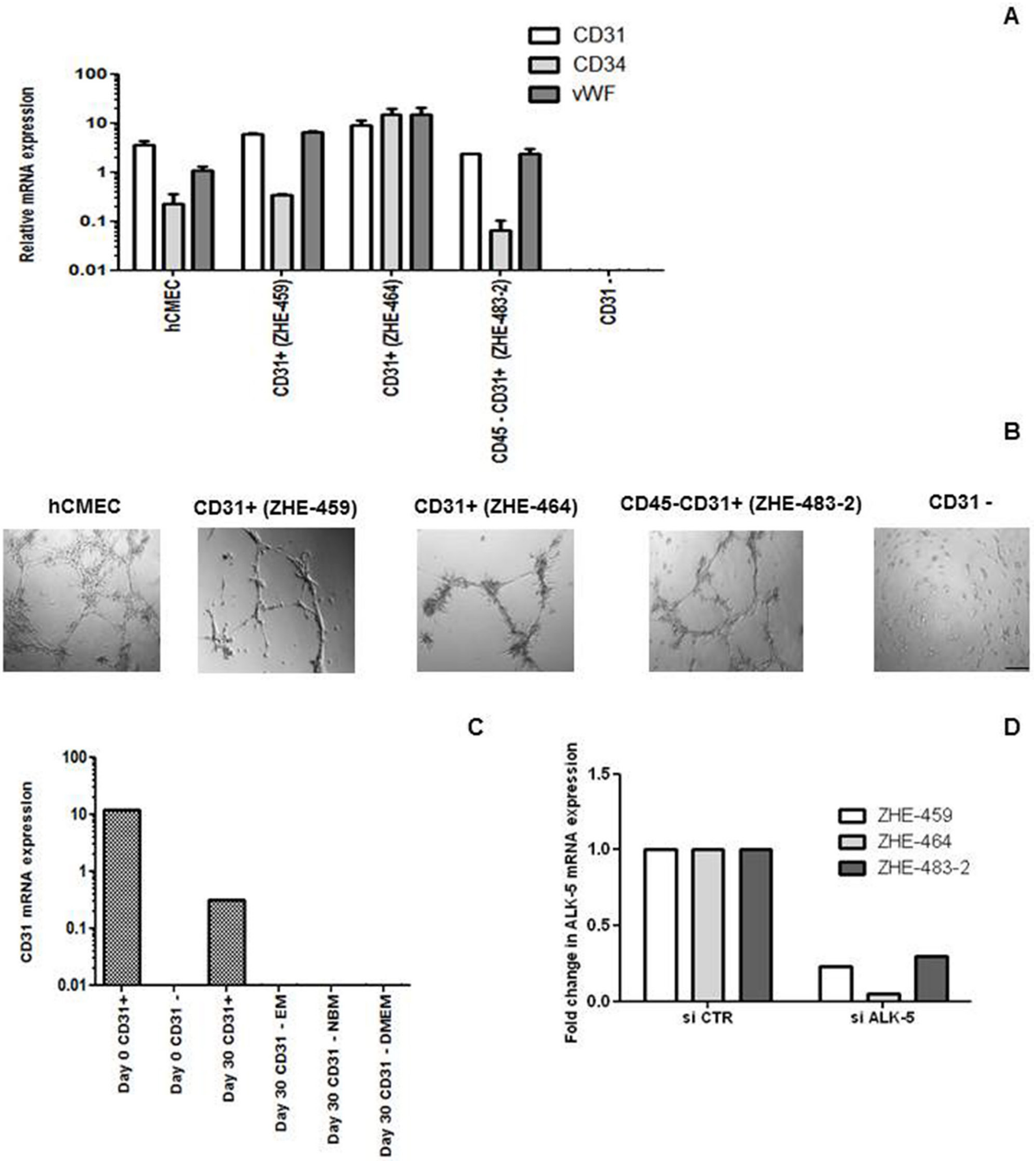

Supplementary Figure 5: Endothelial properties of GMEC. A. CD31-positive cells derived from tumors ZH(E)-459, ZH(E)-464 or ZH(E)-483–2 were assessed for the expression of CD31, CD34, or vWF by qRT-PCR. **B.** ZHE-459, ZHE-464 and ZHE-483–2 CD31-positive cells were starved for 48 h and transferred to growth factor-reduced matrigel and monitored for tube formation. In A and B, hCMEC and CD31-negative cells starved for 48 h were used as positive and negative controls respectively. **C.** CD31 mRNA expression of ZHE-464 cells was determined by qRT-PCR on day 0 (day of isolation) or on day 30 (passage 5) in endothelial medium vs CD31-negative cells cultured in endothelial medium (EM), neurobasal medium (NBM) or Dulbecco's modified eagle's medium (DMEM). **D.** Silencing of ALK-5 was confirmed at the mRNA level after 72 h in ZHE-459, ZHE-464 or ZHE-483–2 cells by qRT-PCR.

Supplementary Table 1: Primers for qRT-PCR

Gene	Primers
VEGF total	FW:5′-GAGACCCTGGTGGACATCTT-3′<br>REV:5′-TTGATCCGCATAATCTGCAT-3′
VEGF-165A	FW:5′-ATAGAGCAAGACAAGAAAATC-3′<br>REV:5′-GTCACATCTGCAAGTACGTTC-3′
VEGF-121A	FW:5′-AAGGCCAGCACATAGGAGAG-3′<br>REV:5′-CTCGGCTTGTCACATTTTTCT-3′
VEGF-148A	FW:5′-CAAGACAAGAAAATCCCTGTGG-3′<br>REV:5′-CCTCGGCTTGTCACATCTT-3′
VEGF-189A	FW:5′-CGGTATAAGTCCTGGAGAGT-3′<br>REV:5′-TCACATCTGCAAGTACGTTCG-3′
VEGF-121B	FW:5′-AGAGATGAGCTTCCTACAGCAC-3′<br>REV:5′-CCTGGTGAGAGATTTTTCTTGTC-3′
VEGF-165B	FW:5′-ATAGAGCAAGACAAGAAAATC-3′<br>REV:5′-CCTGGTGAGAGATCTGCAAGT-3′
PlGF total	FW:5′-CCAGCCACAGCCTTACCTAC-3′<br>REV:5′-GGTGGTTCGAGAATCTTCTG-3′
TβRII	Lot no. 98554236 (Qiagen)
ALK-5	Lot no. 98730849 (Qiagen)
ALK-1	Lot no. 6734902_1855089_88442_88443 (Qiagen)
Endoglin	FW:5′-TCA ACA TGG ACA GCC TCT CTT TC-3′<br>REV:5′-GAC ACT CTG ACC TGC ACA AAG C-3′
TβRIII	FW:5′-TAC AGA GAG AGG TCA CAC T-3′<br>REV:5′-GTC TTC AGA TGC CAC ACC AG-3′
β3-integrin	FW:-CAGATGCCTGCACCTTTAAGAAA-3′<br>REV:-TCACGGCAGTAACGGTTGC-3′
β5-integrin	FW:5′-CGCAATTTGGAAACAACTTGTAAC-3′<br>REV:5′-TGAAAGTCATATCGGATGGCG-3′
vWF	FW:5′-CGGCAACTTTCAAGTCCT-3′<br>REV:5′-GGTCAAGGTCCCTTGTTGGG-3′
CLDN5	FW:5′-GCGTGCTCTACCTGTTTT-3′<br>REV:5′-CAGCTCGTACTTCTGCGA-3′
SP1	FW:5′-GGAAGTGGAGGCAACATCAT-3′<br>REV:5′-TGAGAGCTGGGAGTCAAGGT-3′
EGR1	FW:5′-TGAACAACGAGAAGGTGCTG-3′<br>REV:5′-TGGGTTGGTCATGCTCACTA-3′
SNAI1	FW:5′-CAGTGCCTCGACCACTATGC -3′<br>REV:5′-TGCTGGAAGGTAAACTCTGGAT-3′
N-cadherin	FW:5′-TGGGTCTGTTTTATTACTCCTGGT-3′<br>REV:5′-AGCGAGCTGATGACAAATAGC-3′
ARF-1	5′-GACCACGATCCTCTACAAGC-3′<br>5′-TCCCACACAGTGAAGCTGATG-3′
